# An in-depth study of patent medicine sellers' perspectives on malaria in a rural Nigerian community

**DOI:** 10.1186/1475-2875-5-97

**Published:** 2006-11-01

**Authors:** Theodora A Okeke, Benjamin SC Uzochukwu, Henrietta U Okafor

**Affiliations:** 1 Department of Community Medicine, College of Medicine, University of Nigeria, Enugu-campus, P.O Box 3295 Enugu, Nigeria; 2 Department of Pediatrics, College of Medicine, University of Nigeria, Enugu-campus, PMB 01129 Enugu, Nigeria

## Abstract

**Background:**

Malaria remains a major cause of mortality among under five children in Nigeria. Most of the early treatments for fever and malaria occur through self-medication with antimalarial drugs bought from medicine sellers. These have led to increasing calls for interventions to improve treatment obtained in these outlets. However, information about the current practices of these medicine sellers is needed before such interventions. This study aims to determine the medicine sellers' perspectives on malaria and the determinants that underlie their dispensing patterns of antimalarial drugs.

**Methods:**

The study was conducted in Ugwugo-Nike, a rural community in south-east Nigeria. It involved in-depth interviews with 13 patent medicine sellers.

**Results:**

A majority of the medicine sellers were not trained health professionals and malaria is recognized as a major health problem by them. There is poor knowledge and poor dispensing behaviour in relation to childhood malaria episodes. Although referral of severe malaria is common, there are those who will not refer. Verbal advice is rarely given to the care-givers.

**Conclusion:**

More action research and interventions to improve prescription and referral practices and giving verbal advice to care-givers is recommended. Ways to integrate the drug sellers in the health system are also recommended.

## Background

Malaria is a major public health problem in Nigeria, being one of the five leading causes of out-patient visits and mortality, especially in children under five years of age [1]. Prompt access to early diagnosis and effective antimalarial treatment are major strategies for reducing morbidity and mortality from malaria [2,3].

However, most of the early treatments for fever in most developing countries occur through self-medication with antimalarial drugs bought from patent medicine sellers (PMS) [4-6]. In Nigeria, PMS are usually the first choice in health care and a recognized primary source of orthodox drugs for both rural ad urban populations, especially the poor [7,8]. In addition to selling drugs, they are also a major source of advice about illness and drug therapy [9].

The patent medicine seller can be defined as a person without formal pharmacy training, who sells orthodox pharmaceutical products on a retail basis for profit [10]. Patent medicine stores are owned by the holders of patent and proprietary medicine vendors licenses. Ordinarily the patent medicines should be sold in their original packs. Over-the- counter (OTC) drugs are the only drugs authorized to be sold by the vendors, but they generally sell all types of drugs as determined by their financial capability [11]: these range from paracetamol and chloroquine in large tins to antibiotic, psychotropic drugs, narcotics, toxoids, and antihypertensives, that are outside the scope of their license [12,13]. In addition, the PMS obtain their drug supplies through both formal and informal channels including large retail and wholesale pharmacies in major cities, direct from pharmaceutical companies, and through visiting company representatives [13]. There are reports that these drugs may be ineffective, counterfeit or expired [11].

The Pharmacy Law in Nigeria specifies that PMS should sell only pre-packaged patent medicines. These laws show that the government has positively responded by legislation to forestall a chaotic drug distribution and consumption situation in Nigeria. But evidence suggests that drug laws were adequate, falling short only in their implementation [11].

The PMS have a wide distribution, especially in low income countries, and data on their actual number are difficult to obtain. However, studies show that they are the major drug retailers both in terms of value of drugs sold and number of outlets [14-16].

The reasons for preferring drug shops include geographical accessibility, shorter waiting times, more reliable drug stocks, longer opening hours, greater confidentiality, more personable social interaction, ease of seeking advice, lower cost and flexible pricing policies and no separate fee charged for advice [17,18]. However, one of the problems associated with home management and self medication with drugs from these sellers is that in most cases, neither the drug seller nor the consumer is aware of the correct dosage and duration of treatment [19]. Antimalarial drugs rank high among the drugs administered by these sellers in Nigeria [8]. However, effective management of malaria is not guaranteed: for example, such sellers have been found to consider analgesics containing aspirin as antimalarial drugs [20] and the appropriateness of dispensing in these drug outlets has been found to be suboptimal [21].

These practices may contribute to the spread of chloroquine-resistant *Plasmodium falciparium *in Nigeria [22]. Also, the risks for poor quality treatment may be high, in view of the fact that uncomplicated malaria can proceed rapidly to severe disease and death, especially among young children who have yet to develop immunity [23]. These have led to increasing calls for interventions to improve treatment obtained in these outlets. However, before instituting such interventions, information about the current practices of PMS is needed as well as the determinants that underlie the dispensing pattern of antimalarial drugs in these outlets in Nigeria. While patent medicine shops are acknowledged as a major course of health care products, little has been reported on what actually happens in the shops [10], especially from the shop owner's perspective.

This study aims to determine the patent medicine sellers' perspectives on malaria and their referral practices in a rural Nigerian community. The result, which is part of a larger study designed to ascertain healthcare providers and caretakers' knowledge of malaria symptoms, causes, treatment and referral practices, will help in designing appropriate intervention measures that will help in improving the dispensing behaviour of patent medicine sellers.

## Methods

### Study area

The study was conducted in Ugwogo-Nike, one of the four autonomous communities in Nike, Enugu state, Nigeria. It has a projected population of 13,952 in 2002 and is made up of 10 villages. It has a primary and secondary school, a primary health centre and a comprehensive health centre ('Cottage hospital'), both of which are situated in the same place, but one is managed by local government and the other by state government. Ugwogo-Nike is about 20 kilometres from the state capital, Enugu. This is predominantly a farming community.

Malaria is holoendemic, with a high malaria transmission rate year round and an average malaria incidence rate of 20% [24]. The main malaria vector in Ugwogo is *Anopheles gambiae *and *P. falciparum *is responsible for more than 90% of all malaria infections [24].

A total of 13 patent medicine sellers are registered with the local association. Every four days a large market (in "Orie" market square) attracts many customers from the capital city and neighbouring villages. Market days offer a good opportunity to communicate with community members. Out of the 13 patent medicine sellers, two are located around the market square and 11 are spread out in the 10 villages. Other sources of treatment for this community include 34 traditional healers, the cottage hospital and health centre, and several private and public health care facilities, located in the capital city.

### Study design

The study was a descriptive cross-sectional study. Ugwogo-Nike was selected by simple random sampling from a sample frame of the four autonomous communities in Nike. A census of all medicine sellers was taken by two members of the community with the assistance of the chairman of the local PMS association and a total of 13 were identified. The researchers worked through the chairman of the association to gain legitimacy and support, since PMS are suspicious of outsiders who might be government agents, sent to check them out. Thus, the chairman introduced the researchers to the member PMS and explained the purpose and nature of the study.

### Data collection methods

In-depth interviews (IDI) were conducted with the PMS using IDI guide to explore their knowledge, beliefs, and stated treatment practices for mild and severe malaria, as well as the referral practices for severe malaria. The interview was conducted in the morning hours on non-market days (when the patient flow to the drug sellers' stores was minimal) by two community health nurses from the Health Visiting Unit of the Department of Community Medicine, who had previously been trained in qualitative method techniques. Each interview lasted between 60 and 75 minutes. The interview was tape-recorded and notes taken.

### Data analysis

The transcriptions was organized under thematic headings and later developed into an ethnographic summary with illustrative quotes. The trajectories of their responses to the questions were captured with a trajectory tree, and then the different ranges of opinions, perception and stated practices were noted.

### Ethical considerations

Ethical approval was sought and obtained from the Ethical Committee of the University of Nigeria Teaching Hospital, Enugu (UNTH). The research objectives and methods were explained to individual respondents and verbal informed consent to tape-recording the interviews was obtained from the study participants before the interview commenced. Confidentiality of all information obtained from participants was maintained by not allowing information to be accessible to non-members of the research team.

## Results

All 13 PMS were interviewed. They comprised of eight male and five female practitioners. The age range was from 26 to 42 years. While 10 of them had attained a minimum of primary education, three did not attend any form of formal education. Apart from one nurse who had attended a school of nursing, the highest educational attainment of the sellers was a secondary school certificate and only two had reached this level. All belong to an association of drug sellers, which offers them the opportunity to fraternize together and helps in protecting the members from undue harassment from government agents and community members, as well as maintaining discipline within the association. However, the association does not provide any extra training materials and prescription guidelines to the members. None had any formal health training, except for the nurse who is also a registered member of the Nigerian Nursing and Midwifery council. A male drug seller pointed that *'you don't need to go to a secondary school or university to know how to sell these drugs. All you need is to train under an experienced patent medicine owner for about three years as an apprentice'*.

### Common illnesses affecting children under five years of age

Twelve of the drug sellers believe that, of all the illnesses, malaria is most commonly associated with fever and all affirmed that malaria is the most important of diseases and the one that kills more often. The seller that differed in this opinion was one of the two that had no formal education and, as she put it, *"as far as I am concerned ordinary malaria does not kill children more than diarrhoea, it is diarrhoea that kills the children faster than any other disease in this community"*.

### Causes and mode of transmission of malaria

On the causes of malaria, a few things were predominating in the drug sellers' responses. Some of the PMS have more than one perception about the cause of malaria. Thus, while ten of them felt that mosquitoes were the main cause of malaria, six and four felt that it was due to the sun and drinking bad water, respectively. Less than four sellers also attributed malaria to bad food, cold and body contact. When asked to state how the sun causes malaria, one of them said *"the sun can cause malaria when it shines directly on the child"*. A majority of them believe that mosquito bites transmit malaria, but how it does that was not clear to them. As one of them explained *"if a mosquito bites somebody who has malaria, it transmits his blood to another person when it bites him and he will start to develop Iba" *(*Iba *meaning malaria in the local language).

### Symptoms and signs of malaria

When asked to discuss the symptoms and signs of malaria, they correctly identified them to be fever, headache, weakness and restlessness for mild malaria. According to a female respondent, *'as a mother, I know that once a child has fever and is restless and refuses food, then the child has malaria'*. Others include discoloration of urine and skin, cold, vomiting, loss of appetite, stomach upset, dreaming and excessive sweating. It was noted from the responses of most of the drug sellers that one of the signs indicating that the patient is getting worse is continuous fever and as stated by most of them *'if the child continues to have fever while treatment has gone far then you know the problem is getting worse'*. Some of the drug sellers, however, noted that *'if the child starts jerking (convulsing), it is an indication that the condition is getting worse'*. One of the respondents who had no formal education also noted that in some cases, the mothers describe the symptoms to her and this helps her to classify the malaria. As she puts it *'if the mother says the child has fever that comes with yellow eyes, then I know that the child has serious malaria, but if she says the fever came with only headache and joint pains, I know it is ordinary malaria"*. This respondent, who is a nurse, seemed to be more knowledgeable about symptoms and signs of both mild and severe malaria than any other respondent.

### Treatment practices

When the respondents were asked to say how they make treatment decisions, eight respondents said the customers simply asked for specific medicines, which they provided. According to one male seller, *'when patients come to me, they just say I need medicine for malaria and I give them the antimalarial available in my store'*. Again the quantity and doses of antimalarial drugs sold to clients depends on the amount of money available to the client and these drugs are wrapped in a piece of paper by some of the PMS during dispensing while some claimed to package the drugs in small unlabelled envelopes. A respondent captured it thus: *'at times the patients specify the medicine and the amount of money they have and I sell the medicine to them which corresponds to the amount they have'*. But two of the respondents including the nurse claimed that when patients present an illness complaint, they take history and examine them before recommending any drugs to them. In some cases also, the patients come with a prescription sheet from a doctor and they issue them medicine if available in their store. According to the nurse, *'it is easier for me if they come with a prescription*' while one of the male sellers with primary education said '*if they come with a prescription, I ignore it since I can't read doctors' writing. I will rather ask the patient what is wrong with him/her and treat'*.

On the drugs which they sell to patients, all the sellers indicated a higher use of analgesics/antipyretics (paracetamol/analgin) as an addition to antimalarial drugs because according to most of them *'it reduces the fever fast and helps the antimalarila to be very effective'*. Five of the sellers also felt that the analgesics/antipyretics can cure malaria on its own. As one male seller puts it *'if a customer comes with fever and especially headache, I just give them novalgin injection and the fever and headache disappears'*. Eleven of the sellers will use chloroquine as their first line drug administered as injection. When asked the reasons for the use of chloroquine, most of the sellers said chloroquine is the cheapest antimalarial and the patients can afford it. Also eleven of the sellers felt it is still very effective in treating malaria. Only one out of the thirteen interviewed believed halofantrine is more effective and uses it if patients can afford it. According to him, *'this chloroquine don't work any more, so when my patients come, I tell them about Halfan *(Halofantrine) *and if they can afford it, I sell it to them otherwise I give them chloroquine or Fansidar' *(sulfadoxine/pyremethamine, SP). All the sellers had some knowledge of SP preparations and one of them prefers it because some patients believe '*it is more effective than chloroquine and does not cause scratching'*.

For what is assumed to be mild malaria, ten of the sellers will dispense chloroquine tablets and syrup to patients and ask mothers to give it to the child three times in a day, for a period of 3–5 days and sometimes extending to more than five days. But when chloroquine injection is used, the medicine seller administers it him/herself once a day in combination with vitamin B complex injection. According to some of them, *'vitamin B complex helps to build up the blood which malaria infection have consumed'*. However three of the sellers felt differently. According to one of them, *'I give vitamin B complex so as to increase my bill'*. When asked why they administer injections, they claimed it was more effective than the tablets and the syrup and in some cases, it is due to the demand of the patients. This was captured by one of the male sellers thus *'you know injections work faster and at times the patients demand that we give it to them'*. Another respondent stated that *'to make our charges higher, we have to administer injections as this is the only way they can respect you'*. They also claimed that, at times, they dispense these drugs according to the amount of money the customer came with.

Nine of the sellers will recommend the same dosage of chloroquine to all children less than five years of age. Also, some will recommend a single dose of chloroquine for children less than five years old, instead of the required three-day treatment. When reminded that all these might be an over-dose or under-dose for the children, one of them insisted that *'I have been doing this for a long time and they have been recovering without any problem'*. The drug seller who prefers "maloxine" a brand of SP would not change to another antimalarial even if symptoms persists because according to him *'the illness is no more malaria'*. For severe malaria, they indicated that they make use of chloroquine and analgesics (paracetamol) as major drugs while administering vitamin B complex injection if there is need. Other drugs they make use of though less frequently are analgin injection (antipyretic), maloxine (a brand of SP), maloquine (mefloquine), halfan (halofantrine) and multivitamin tablets. Very few indicate their use of blood tonics and drips (intravenous infusion). Three of the PMS interviewed claimed that they rely on the instruction provided by the manufacturers in selling and dispensing the drugs.

Although all the respondents have heard of artemisinin derivatives, none stored them because as they claimed *'we cannot stock because of the high cost. If you do, no villager will buy it from you because they are very poor'*. However, the respondent who stored halofantrine said he cannot afford to store two expensive antimalarial drugs at same time, but claimed that he has on two occasions, bought it for a client who requested it. And on those occasions, the client was said to have got better faster. As he put it *'there is one woman who is always requesting for coartem (ACT) and each time, she comes back to say the drug is wonderful but expensive'*. The respondent said he would be willing to store the drug if there is a demand for it. Apart from prescribing and dispensing drugs, most of them indicated that they will also tepid sponge the child to bring down the temperature. When questioned on why they have to prescribe and what guides their treatment decision, the response from most of the drug sellers was that they have the experience. For the convulsing child all claimed they will refer to a health facility. Two of them will tepid sponge and give antipyretics before referral, while one drug seller will give an anti-emetic promethazine or administer an anticonvulsant "phenobarbitone" before referral.

Only five of the sellers will communicate to the caregiver the dosage of the medicines and none of the sellers will communicate the precautions and side effects of the medicines purchased by the patient.

### Referral practices

On referral practices, most of the sellers hardly refer patients to the health centre or the cottage hospital in the community because *'the workers are hardly ever there and there is no doctor in the places'*. However, three of the drug sellers claimed they would refer patients to the cottage hospital for the treatment of mild malaria, but if the child develops convulsion, nine of them will refer to a doctor in the city. The nurse claimed that she refers all cases not responding to antimalarial drugs to a doctor in the capital city. As she put it *'any malaria I cannot treat here cannot be treated at the health centre. So I have to refer them to a doctor in the urban'*. For those who will refer, they were asked to state the mode of referral. Only the nurse said she will give the caregiver a referral note to the facility. The rest will only ask the caregiver to go elsewhere verbally. A few, however, indicated they will not refer, but rather treat the cases by making use of paracetamol or novalgin, (analgesics/antipyretic), paraldehyde (anticonvulsant) and promethazin (anti-emetic) injections. As put by one of them *, 'I am very good at treating malaria, so I don't need to refer to anybody. When you refer, the mothers will think you don't know anything and will not come another day'*.

## Discussion

This study documents the knowledge and stated practices of medicine sellers for the treatment of childhood malaria in a small rural community. The results show that only one drug seller had a health training background. This is a very low figure and contrasts with studies done in Uganda, where a majority of them had one form of health training or another [25]. The PMS's educational level is usually not specified in laws governing licensing of such practice although by convention, as is the case in Nigeria, the minimum educational attainment has been primary schooling [26]. These sellers have been known to enter the business from a variety of backgrounds unconnected with having adequate knowledge of illness perception and management, making effective malaria control impracticable [27]. Although having a health education background is not required for registration of their premises, having no form of health training is likely to contribute to inappropriate dispensing of antimalarial drugs, especially where some of the respondents do not have even a primary education.

Although the respondents were generally well informed about symptoms and causes of malaria, there were some misconceptions about the causes of malaria, incriminating the hot sun and drinking bad water. This perception is likely to pose problem when taking decisions on what drugs to give. It has been noted that knowledge of the causes of disease and their symptoms and signs is important guide in determining the choice of drug treatment [25]. The fact that the nurse was more knowledgeable than the rest underscores the need for the sellers to have either formal or informal medical knowledge.

In this study, chloroquine is still commonly used and inappropriately too by the drug sellers in conjunction with antipyretics/analgesics (paracetamol and analgin). This undermines therapeutic efficacy and may promote the emergence and further spread of drug-resistance. In Nigeria there has been a change in the national antimalarial drug policy to artemisinin-based combination therapy (ACT) as a first line drug for the treatment of uncomplicated malaria because of chloroquine resistance. In reality, access to ACT is still very poor and most cases of malaria are still treated with chloroquine by the PMS.

Furthermore, chloroquine injections were administered commonly, a finding consistent with earlier studies in Nigeria [28,29]. The use of chloroquine injections may be an indication that sellers are primarily motivated by their need to make profits, and not by any wish to provide a public health good. It has been noted that prescribing patterns are more likely to follow patient demands and expectations and profit motive rather than professional principles. There is concern about the frequent administration of injections by individuals with no health background especially in light of the high risk of transmitting HIV and other blood-borne infections [30]. Thus training alone is unlikely to deal with the issue. Hence the need for appropriate monitoring and media campaign by government. The current effort in Nigeria by the National Agency for Food and Drug Administration and Control (NAFDAC) on dangers of unnecessary injections through mass media campaign is a step in the right direction.

Although a good number of the vendors had adequate knowledge of signs and symptoms of malaria, the results show that their knowledge of correct antimalarial dosage is low for both mild and severe childhood malaria leading to the administration of both over- and under-dosage of antimalarial drugs. This same phenomenon was noted elsewhere [31]. The ineffectiveness of these practices represents a major problem, since many malaria deaths occur within the first 48 h of illness [23]. The great danger with under dosage is also the development of chloroquine-resistant strains of *P. falciparum *which has reduced the efficacy of chloroquine in Nigeria [32].

Analgesics/antipyretics are always prescribed in this study with chloroquine. Some of the medicine sellers appear to confuse analgesics/antipyretics with antimalarials. This is a common phenomenon and had previously been noted in Nigeria [20].

Halofantrine is still being used by some of the drug sellers. The cardio-toxicity effect of halofantrine has been documented [33] and this drug has been banned by some countries because of this. However, it is interesting to note that the sellers are knowledgeable about ACT. It will be worthwhile to explore this in the wake of changing antimalarial first line drug policy in Nigeria to see the possibility of making ACT available to consumers through the PMS in the spirit of public-private partnership.

Although referral of severe malaria cases was common, little or no verbal advice is given to mothers and no formal arrangement is made to facilitate the referral. This could be improved through training of both mothers and the PMS on recognition of conditions warranting referral and establishing a network between the PMS and the formal health sector as well as the local transport union to ensure easy accessibility.

## Limitation of the study

The study involved only 13 drug vendors in a small area. However, there has been a recent surge of international interest in health-related small area studies and the World Bank is encouraging the use of small area analysis [34]. The results of this study may reflect what is happening in rural areas of Nigeria because it has all the features of a typical Nigerian village. Furthermore, the results compares with other studies in Nigeria [10,13] and will be useful in guiding intervention studies targeted at rural communities and the health sector and thus reduce the effect of malaria.

## Conclusion

This study brings up the issues regarding the poor knowledge and poor dispensing behaviour of PMS in relation to childhood malaria episodes. It has been shown that most of the drug sellers were not trained health professionals and malaria is recognized as a major health problem by most of them. However, there are some misconceptions on its causes and PMS are currently not very good at providing appropriate advice and adequate doses of antimalarial to their clients. Training and periodic re-training of drug sellers to enable them provide correct and full therapy for uncomplicated malaria, appreciate the importance of giving verbal advice to care-givers and recognize and refer promptly all severe malaria cases is recommended. The effectiveness of such trainings has been recorded in different settings [23,35-37].

PMS knowledge about product dosages and precautions could also be improved with the provision of better product description by pharmacy companies and the pre-packaging of antimalarials for young children. This has been found to be effective in Nigeria [38].

There is need for sustainable monitoring systems. Monitoring and influencing the quality of private services is now recognized as a key component of effective malaria treatment [39]. The PMS union can be used to monitor themselves when trained and this novel idea will be tested during the intervention phase of this study. At present, the PMS are registered and monitored by the state Ministry of Health, Pharmacy division.

## Competing interests

The author(s) declare that they have no competing interests.

## Authors' contributions

AO conceived and designed the study. All the authors participated in data collection and analysis. BU wrote the manuscript with input from all the authors.
